# Neurosurgery Trainees’ Perspectives of the ‘Mock Viva’

**DOI:** 10.15694/mep.2018.0000248.1

**Published:** 2018-11-07

**Authors:** Grainne McKenna

**Affiliations:** 1 University College London

**Keywords:** Formative assessment, mock viva examination, surgical training, neurosurgical training, simulation

## Abstract

This article was migrated. The article was marked as recommended.

The ‘viva voce’ examination has been a tradition in surgical training for over a century, and remains a key element of the ‘Fellowship of the Royal College of Surgeons’ (FRCS) Examination, which neurosurgery trainees must pass to complete their training. The aims of this study were to evaluate the educational value of an annual ‘mock viva’ as a formative assessment tool for neurosurgical trainees during their eight year training programme, to identify barriers to participating in the mock viva and to explore how these might be attenuated to increase levels of engagement **.**

A mixed deductive and inductive methodology was employed for the study design and qualitative data analysis. Semi structured interviews were conducted with two cohorts of trainees (three pre-FRCS and three post-FRCS).

Six overarching themes emerged from thematic analysis of coded qualitative data – ‘insight’, ‘performance’, ‘simulation’, ‘stress’, ‘differentiation strategies’, and ‘assessment versus learning’.

Gaining insight into the format, standard, marking scheme, and processes of the final FRCS examination were considered key elements of the educational value of the mock viva and these were perceived to be acquired through high fidelity simulation of the FRCS exam, with high quality feedback on performance. The opportunity to observe the assessment of others offered insight into one’s performance relative to peers as well as insight the perspective of the examiner conducting the assessment.

Whilst they acknowledged negative ‘stress’ factors associated with the mock viva, post-FRCS trainees underscored the benefits of learning from substandard performances; of reflecting on these experiences and on the feedback they received, and they suggested that junior trainees lacked insight into the educational value of what are often perceived to be ‘negative’ experiences. Junior trainees were concerned that the potential learning value of the mock viva process was limited by their clinical knowledge and experience in the early stage of training.

These overarching themes point to areas which could be targeted to enhance the educational value of the mock viva and to address the paradox of poor uptake amongst junior trainees.

## Introduction

Neurosurgery trainees take the Intercollegiate FRCS examination at the end of their training, which requires them to demonstrate knowledge and clinical competence commensurate with that of a newly qualified consultant neurosurgeon. One part of this summative process is the ‘Oral Examination’, which is a structured ‘viva voce’ designed to test knowledge, higher order thinking and decision-making skills.

The overall pass rate for the examination is 50-60% (JCIE Results, 2015-17). There is speculation that it is not lack of knowledge per se which is responsible for the low pass rate, but rather ‘poor confidence, which translates into weak delivery and hesitant performance’ (Choi, in Elwell et al, 2015).

It was with this principle in mind that an annual ‘Mock Viva’ was established in my deanery, with the aim of practicing the oral examination in a formative setting throughout the training programme, thus improving trainees’ performance in the final summative examination.

Whilst there appear to be clear advantages to ‘rehearse’ in the run-up to the final exam, the take-up rate for the annual mock viva amongst the cohort of neurosurgery trainees in my deanery is often lower than expected, particularly amongst junior trainees.

There is a small body of research from the USA demonstrating perceived advantages and modest objective improvements in first time pass rates for equivalent postgraduate surgical examinations with the implementation of mock oral examinations ( Aboulian et al., 2010; Fingeret et al., 2016; Smeds et al., 2018).

Beyond the primary objective of improving final pass rates, some evaluative studies have shown additional perceived benefits of mock oral examinations, including improvements in clinical reasoning, promotion of self-study and development of professional and communication skills ( Higgins et al., 2016; Pennell and McCulloch, 2015). These studies, although of limited quality, are based on educational settings closely related to the one in question.

Compounding the challenge of performing well in these high-stakes summative assessments is the inherent ‘stress’ factor. Although there is evidence that higher levels of stress can improve performance, research has shown that performance increases only up to a point and, paradoxically, for complex tasks, if levels of arousal become too high, performance actually decreases. This is thought to be due to negative effects of stress on cognitive processes like attention, memory, and problem-solving ( Yerkes and Dodson, 1908).

Thus, it follows that to be prepared for the oral examination candidates need to be performing at the level of ‘proficient’ or ‘expert’ in the Drefus model of adult skill acquisition ( Dreyfus and Dreyfus, 2004), to accommodate the negative impact of stress.

### Context of Study

To investigate the paradox between the promising aims of the mock viva and surprisingly poor engagement with the process, I decided to evaluate trainees’ perceptions of the viva process.

As a senior neurosurgical trainee, who has recently completed the FRCS examination, I effectively have a ‘foot in both camps’, as both an exam candidate who has experienced first-hand the challenge of the FRCS viva, and in my new role of co-organiser and mock examiner in the next annual formative oral examination. Inevitably, I have my own views on this particular formative assessment; and whilst my new role as an examiner may bias the responses from some participants, it also gives me the opportunity to address any areas for improvement which may become apparent.

Permission was granted from my Training Programme Director to recruit participants from the current cohort of neurosurgical trainees.

### Study Question

What is the educational value of the mock viva to neurosurgical trainees, and how can it be enhanced?

### Objectives

• To explore trainees’ perspectives on the value of the mock viva as an annual formative assessment tool in early years training.
• To explore the barriers or potential down-sides to participating in the mock viva and how they could be attenuated to increase levels of engagement.
• To compare the perspectives of trainees who are pre and post-FRCS examination on the utility the mock viva for preparing for the summative final examination.

## Methods

I investigated the perspectives of two separate cohorts of trainees, because I hypothesised that trainees pre- and post-FRCS would have different views on these topics based on their different experiences, and using a deductive approach, I anticipated that triangulating the data collection from different groups would be key to answering my research question.

The first cohort of pre-FRCS neurosurgical trainees (ST1-6) were investigated with a series of semi-structured individual interviews. I had intended to use a focus group for this cohort of trainees with the expectation that building on the ideas of other participants through facilitated discussion would produce richer data. However, this was not feasible to arrange and I opted to conduct individual interviews instead. This strategy offered the advantage of anonymity and reduced the effect of bias in the responses. In order to build on the ideas of others and find out to what extent these perspectives were shared by the pre-FRCS cohort, I presented anonymised comments and ideas generated from earlier interviews to the remaining participants, in order to canvas opinion on these theories.

The second cohort of post FRCS trainees (ST7-8 or equivalent) were also investigated with semi-structured individual interviews. This method of data collection was chosen in preference to a focus group to protect the anonymity of potentially sensitive personal information, and to avoid conflict of interest, particularly in the context of some participants who had multiple attempts at the summative examination, or had yet to achieve a pass. Whilst this was time-consuming, it facilitated in depth, personal responses from participants, with less risk of bias from others.

I invited trainees from my own department to participate in the interviews, partly selected by convenience, but also because my department has within it a suitable range of junior and senior trainees to enable me to answer my study question. I selected three trainees for each group with the aim of obtaining a range of opinions and ideas, without compromising the efficiency and practicality of the data collection.

Interview questions for the first cohort were tailored with an inductive approach and were firmly rooted in the evaluation aims and in the expectation of generating new theories from the data (Appendix 1).

The data from the interview transcripts were coded and a thematic approach was used for analysis of the results. Codes from the analysis of the pre-FRCS cohort data were identified and used to construct a framework for the second cohort of interviews with post-FRCS trainees. Thus, a mixed deductive and inductive methodology was employed for my study design and qualitative data analysis.

This study had no direct relevance to patient care. Interview transcripts were anonymised and individual responses were kept confidential when discussing newly generated ideas with other participants.

As a senior trainee, I could relate to participants in both cohorts and as an interviewer I was able to facilitate a deep discussion about the training issues in a supportive and non-judgemental environment. However, the impact of my relationship as a fellow trainee could be confounded by my role as a mock examiner and could have influenced the responses from participants.

To ensure that these potential concerns were recognised and addressed, trainees were given an information sheet on the study and were asked to sign a consent form prior to participation (Appendices 2 & 3).

The final report was shared with my Training Programme Director (TPD), so any significant findings arising from the study could be highlighted for review.

## Results/Analysis

There were six participants, as outlined in Table 1, who were interviewed in the following order.

**Table 1.** Participant Demographics

Participant	Level of training	FRCS exam status
P1	ST3	Pre-FRCS
P2	ST3	Pre-FRCS
P3	ST4	Pre-FRCS
P4	ST8	Post-FRCS
P5	ST8	Post-FRCS
P6	ST8	Post-FRCS

Six clear overarching themes emerged from thematic analysis of coded qualitative data from the interview transcripts, as outlined in Table 2.

**Table 2.** Thematic Analysis

Code references (Post FRCS participants in bold)	Sub-themes	Themes	Overarching Themes
P1; P3	Structure	Exam format	**Insight**
P1; **P5**	Marking Scheme		
P3; **P5**	Classic exam cases		
P3; **P6**	Usefulness of feedback	Frame of reference for performance	
P1	Gaining perspective of examiner by observing others		
P1; P2; P3	Level of knowledge relative to peers		
P1; P2; P3	Standard required for FRCS		
P5	Limitations of feedback and frame of reference		
P1; P2; **P4**	Forces acknowledgement of unknowns		
**P4; P6**	Lack of insight into what can be gained from the process of the mock viva	Junior trainees lack insight	
	Lack of insight into their learning needs in the approach to the FRCS exam.		
P2; **P5;** **P6**	Convincing performance		**Performance**
P2; P3	Confident performance		
P1; P2; **P4:**	Practiced performance		
P1; **P5;** **P6**	Developing a system	Exam technique	**Simulation**
**P4; P6**	Increases likelihood of success in FRCS exam	Practice	
P2; P3; **P5**	Rehearsal		
P1; P2; P3	Independent assessors	Must be high fidelity simulation of exam	
P3	Vary the examining style		
P2; **P4; P6**	Daily viva sessions in handover meetings as a panacea to the problems of the annual mock viva	Frequency of simulation	
			
**P4; P5; P6**	Learning from failures	Challenge	**Stress**
P3; **P4**	Rising to challenge and competition		
P3	Time pressured		
P1; P2; P3; **P4; P5; P6**	Pressure as a negative factor	Anxiety	
**P4; P5**	Pressure as a motivating factor		
			
P1; P2; **P4; P5; P6**	Limited knowledge limits educational value of mock viva process	Content vs Process	**Differentiation strategies for junior and senior trainees**
P1; P2; P3; **P5; P6**	Engaging with the viva process is possible and necessary at the early stages of training, independent of level of knowledge		
P1; **P4; P6**	Paradox of poor delivery in context of good knowledge		
P2; **P6**	Support for coaching	Coaching for junior trainees	
P3; **P4**	Against coaching		
P1; **P5**	Learning the ‘hidden curriculum’	Learning opportunity	**Assessment versus Learning**
P1: P3	Practice rather than learning		
P1	Assessment detracts from the learning opportunity	Assessment	
P2	Primary aim is assessment		
P2; **P4; P5**	Assessment promotes learning		
P2; **P5**	Implications for future training	Judgement	
P3	Opportunity to demonstrate competence		

Code references and full interview transcripts are listed in Appendices 4 and 5 respectively.

Comparison of responses from the two cohorts of participants revealed some notable differences. Whilst all the participants reported negative effects of stress and anxiety on their performance in the mock viva, it was only the post-FRCS participants who reported the educational value of learning from these ‘failures’ in their mock exam performances, and they did so unanimously.

As one post-FRCS participant described, “The best mock exams were when my knowledge was pushed to the very limit… Those exam scenarios stuck to my mind the best, so in fact, although it may not feel like it at that moment in time, you learn most from that and you might actually benefit the most from those” (P4, Appendix 4).

This senior cohort of trainees also exclusively reported the positive effect of stress as a motivating factor in promoting learning, and the role of the mock viva in increasing the chance of success in the FRCS exam.

In their responses, the post-FRCS participants all heavily emphasised the importance of the mock viva in helping them to develop a system for answering questions, which emerged as the theme of ‘exam technique’. They also emphasized the ‘performance’ nature of the exam and the need to develop a confident delivery such that one presents oneself to examiners as a credible colleague. As one senior trainee summed up, “You are being judged by consultant colleagues based on this performance, and so how have to convince them that you are one of them” (P5, Appendix 4).

The concept of developing ‘insight’ into the exam process, the standards and one’s relative performance was a repetitive theme in the responses. Whilst all the participants described the general benefits of finding out about the process and the standards, the post-FRCS cohort offered a more nuanced perspective on this topic. They felt that junior trainees were failing to appreciate the immense educational potential of the mock viva, and that their failure to overcome their anxieties about the process was counterproductive, as it is an essential process in preparation for the exam. They emphasised the importance of engaging with mock viva examinations early in training, so they can learn more about what they do *not* know. In describing this lack of insight in junior trainees, one post-FRCS participant pointed out, “If you know less, then this awareness of ignorance is less likely to be there” (P4, Appendix 4).

Participants from both cohorts argued that limited levels of knowledge and experience in the junior trainees, meant there was little to be gained from their participation in the mock viva as they had insufficient ‘content’ to meaningfully practice the ‘process’. Other post-FRCS trainees argued that the system can be learned on the most basic of clinical knowledge and should be put into practice early in training.

This perceived deficiency in ‘content’ was the source of many anxieties surrounding the mock viva, and fed into another overarching theme of whether the mock viva represented an ‘assessment’ or a ‘learning’ opportunity. As a formative assessment its function was perceived to be dependent on the quality of feedback given, and the learning experience of the trainee as an observer of others. Junior trainees reported value in comparing their performance with those of peers and from observing their performance in an imaginary role as an examiner (P1, Appendix 4). However, a number of trainees reported stress associated with judgement from peers and consultant colleagues, which could impact their future training, and felt threatened by the potential conflict of interest.

From these overarching themes a number of ideas for enhancing the educational value of the mock viva experience were developed. These included differentiated approaches to formative assessment for junior and senior trainees, such as coaching in viva preparation for juniors, and pre-defined ‘content’ for juniors, so that the focus of their viva is on simulating the ‘process’. Other suggestions for improvement arose from themes which highlighted the importance of high fidelity simulation, such as recruiting independent assessors from different units to vary the questioning styles and the case scenarios, and to eliminate bias or conflicts of interest.

## Discussion

These six overarching themes highlight the complexities of learning a hidden curriculum through formative assessment and point to areas which could be targeted to enhance the educational value of the mock viva.

Participants from both cohorts described the exam as a ‘performance’, and suggest that one needs to learn how to ‘play the game’. This points to a hidden curriculum ( *Jackson*, 1968) which must be learned. And as one post-FRCS participant described, “No one teaches you that. It’s trial and error” (P6, Appendix 5). This concept reflects expert opinion that “Demonstrating competence is ultimately a ‘performance’ – involving ‘a plethora of soft skills’” (Elwell et al, 2015).

Gaining insight into the format, marking scheme, processes, and one’s performance relative to peers and the final FRCS standard are all considered key elements of the educational value of the mock viva and are perceived to be acquired through high fidelity simulation of the FRCS exam, with high quality feedback, and the opportunity to observe peers, which offers insight into performance from the perspective of the examiner. These perceived benefits are grounded in experiential learning theory and add to evidence from other studies which suggest that conducting mock oral exams in a public forum can add educational value for observers, even if they are not preparing for a summative examination process themselves ( Aboulian et al, 2010).

Whilst they recognised the negative stress factors associated with the mock viva, the post-FRCS cohort also pointed to the benefits of learning from poor performances; of reflecting on these experiences and on the feedback they received, and suggested that junior trainees lacked insight into the educational value what are perceived to be ‘negative’ experiences.

This concept mirrors the educational theory of experiential learning described in Kolb’s learning cycle ( Kolb, 1984) and emphasizes the importance of learning through the iterative process of reflection on experiences and of developing theories and action plans to improve performance. Repetitive simulation of the exam in a formative setting is likely to reduce the complexity of the task and thus will offset the heightened levels of arousal in the summative exam, which can have negative effects on cognitive processes like attention, memory, and problem-solving as postulated by Yerkes & Dodson (1908).

This concept is also reflected in petitions for routine viva practice as part of a daily handover meeting. It was felt that this would be a panacea for addressing the issues of ‘content’ and ‘process’ simultaneously, and facilitate differentiated instruction for heterogeneous groups of trainees. The strategy of frequent simulation “to acclimatise to the stress”, by being “quizzed regularly by colleagues or senior surgeons, to experience the adrenaline rush of being ‘put on the spot’ and to become accustomed to answering questions under pressure” is also advocated by exam expert David Choi (Choi, in Elwell et al, 2015).

The theme of developing more insight as one progresses from novice to expert, and ‘unknown unknowns’ become ‘known unknowns’ is also grounded in the Drefus model of adult skill acquisition ( Dreyfus and Dreyfus, 2004) and is, perhaps unsurprisingly, strikingly reflected in the official scoring sheet of the Examination Board (JCIE Marking Descriptors, 2016).

### Recommendations for local practice

• Introduce coaching in viva preparation for ST1-2 trainees
• Limit the content to be covered in the mock viva as appropriate for each stage of training and allow preparation of this predefined content in advance of the session
• Recruit independent examiners to eliminate conflict of interests and increase fidelity of exam simulation
• Introduce routine daily viva sessions which will create opportunities for differentiated instruction and for trainees to observe and reflect on the performance of others
• Train examiners in giving effective feedback

Areas for future investigation include implementation of these strategies and evaluation of their effectiveness.

### Limitations

This was a small evaluative study and my role as both a fellow trainee and mock examiner had the potential to introduce bias into the questioning, the responses from participants and the data interpretation. However, the two sample cohorts of trainees are strongly representative of the body of trainees in my deanery and consistent themes have emerged from the data analysis which are convincing and generalizable to the study area.

Effective implementation of these recommendations will require a cultural change within the educational setting and this calls for strong leadership and engagement of all the stakeholders in the mock viva process.

## Conclusion

This study has demonstrated a wealth of perceived educational benefits of participating in the mock viva, and has shed light on the factors which contribute to the paradox of poor uptake amongst junior trainees. Exploration of the themes which emerged from the data analysis, generated strategies for attenuating the negative stress factors, increasing levels of engagement and differentiating the instruction for junior and senior trainees to enhance the educational yield of the mock viva process.

## Take Home Messages

• Trainees perceive the mock viva to offer a wealth of potential educational value, through high fidelity simulation of the FRCS exam, high quality feedback, and the opportunity to observe peers, which offers insight into performance from the perspective of an examiner.
• Whilst junior trainees perceived poor performances to be ‘negative’ and stressful experiences, post-FRCS trainees had a strong appreciation for the educational value of receiving critical feedback and reflecting on substandard performances.
• The educational yield of ‘mock vivas’ could be enhanced using differentiated approaches for junior and senior trainees to alleviate stress factors and increase levels of engagement.

## Notes On Contributors

Gráinne McKenna MA(Cantab) MB BChir FRCS(SN) is an ST8 neurosurgical trainee in London and is undertaking an MSc in Medical Education with UCL and The Royal College of Physicians.

## Figures and Tables

**Table:** 

Code references	Sub-themes	Themes	Overarching Themes
P1: Structure; Format; structure answers; rigid; P3: Get into the habit of thinking in the framework that is necessary for the exam	Structure	Exam format	**Insight**
P1: See the mark scheme P5: getting familiar with the mark scheme,	Marking Scheme		
P3: it’s useful to be acquainted to those sorts of cases P5: gaining insight into the classical exam scenarios	Classic exam cases		
P3: The feedback at the end from the examiners is probably one of the most important bits of it. P6: *quoting a UHT neurosurgeon (Sampson) -* the first application of the clip will show you what you need to do, and it guides the way for the second aneurysm application. *(ie. First attempts give valuable and necessary feedback to achieve final success)*	Usefulness of feedback	Frame of reference for performance	
P1: You can also imagine yourself as an examiner, Useful to realise how important it is how you come across to the examiner.	Gaining perspective of examiner by observing others		
P2: level of content P2: not on par with people in your same peer group P3: level of knowledge relative to peers; competitive comparison	Level of knowledge relative to peers		
P1: Helps identify what you need to know P2: gauge how much preparation you need to do P3: gives me an idea of what my knowledge level is like in comparison to what it’s meant to be at the end	Standard required for FRCS	Frame of reference for performance	**Insight**
P5: It all depends on the quality of the feedback, and often trainees measure their own performance on the basis of comparison with just one of their peers, which isn’t really a useful benchmark for what they need to know long term; there are no real scores or measure of performance relative to the whole cohort. In other units, you need to have been assessed to have passed a formative viva locally before you will be supported to apply to sit the FRCS, and so trainees take it very seriously. Ours is so informal, no one really prepares for it or treats it like a real exam.	Limitations of feedback and frame of reference		
P1: Shatters any delusions P2: Eye-opening P4: again if you push to the area where you give wrong answers, and you are aware it is wrong, and the feedback is not as good as expected, well, that is also good, so then you will know what you don’t know.	Forces acknowledgement of unknowns		
P4: Some think they will be embarrassed even if their line of thought is good; P4: In 2 or 3 more years they will be in more senior positions and the time wasted now is time wasted forever. So they will have to catch up fairly rapidly when the time comes at some point. And then it will be really stressful. So I think these fears are subjective and they are completely irrational. P4: If you know less, then this awareness of ignorance is less likely to be there. P6: In agreeing with interview that .. *everything that you have described there is something that I think trainees lack insight into.*	Lack of insight into what can be gained from the process of the mock viva	Junior trainees lack insight	
	Lack of insight into their learning needs in the approach to the FRCS exam.		
P2: persuade two or three consultant neurosurgeons that I know what I’m talking about. P5: The viva exam is a performance. You are being judged by consultant colleagues based on this performance, and so how have to convince them that you are one of them. P6: the mind set is different from the written exam, and you are marked on how fluid and calm you are, and that comes across to the examiners straight away; there’s nowhere to hide in the viva. You need to have a poker face.	Convincing performance		**Performance**
P2: can either boost your confidence or knock your confidence; then it is just the content that is going to make you feel not confident, which will impact on your performance. P3: perform in front of their peers	Confident performance		
P1: Formal practice P2: Reacclimatised P4: It is simply practice giving an answer and thinking through the cases	Practiced performance		
P1: Artificial learning for exam P5: Most of this we do on a day to day basis, but the exam is a highly pressurised situation, and so you have to practice the ‘exam technique’ in order to perform well on the day. P6: being able to figure out what the examiner wants and give it to them in a nice package, so to speak. So understanding what they want from the question; being able to shift between different aspects of concepts in the middle of the exam. P6: viva teaches you how to condense that into relative things, the important things that you can deliver in 2-3 minutes. You know, it will be 20-30 pages in a book, but there are key points that examiners want you to touch upon and from those key points they will decide on whether you have a sound understanding of the topic or not. So getting from 20 pages in Greenberg to a very nice, sophisticated, compact, concise delivery in 2 minutes, that’s difficult, you know, no one teaches you that. It’s trial and error	Developing a system	Exam technique	**Simulation**
P4: it’s essential and I still think that the more mock viva experience one has, the better the chances of passing P6: So it’s like top gun or desert flag for neurosurgery trainees; after the Vietnam War, they found that pilots who survived the first 10 missions were much more likely to survive the rest of the war. And that’s why they aimed to simulate the first 10 missions.	Increases likelihood of success in FRCS exam	Practice	
P2: Reacclimatised to exam P3: if you cover most of them in this rehearsal setting, then it is unlikely you will go to the exam and be phased by something you have totally not thought about at all. P5: Towards the end, getting in the zone, or exam mode, where you are imagining it is the real thing, so that you are familiar with the format and the feeling of being put on the spot on the day; It’s important to experience a crash and burn station, so that you learn to pick yourself up and keep going with the rest of the exam, so you don’t blow the whole thing.	Rehearsal		
P1: Realistic to be of benefit; Artificial learning for exam, not reflective of real life P2: consultants could be brought in from outside P3: people that you are less close to and therefore a bit more like a real exam in terms of you not knowing the examiners, and them not knowing you	Independent assessors	Must be high fidelity simulation of exam	**Simulation**
P3: it’s the same people running it, and so they will repeat the cases that they know about, so I guess having more variety would be more helpful; different places with different examiners, so there’s a bit more variety in examining styles.	Vary the examining style		
P2: a lot of that training is done more on delivery of content than anything else P4: Daily questioning is essential. If you start doing is regularly, even coming in early to do so, after six months or so, you really start going into greater depth.. So if you do that consistently, then at the end of six months, you can say we are going to wrap this up in a more formalised way. P6: more continuous preparation for the viva, with more ‘hot spotting’ in handover meetings and so forth.	Daily viva sessions in handover meetings as a panacea to the problems of the annual mock viva	Frequency of simulation	
P4: the best mock exams were when my knowledge was pushed to the very limit, and when I could not produce coherent and adequate answers. Those exam scenarios stuck to my mind the best, so in fact, although it may not feel like it at that moment in time, you learn most from that and you might actually benefit the most from those; again if you push to the area where you give wrong answers, and you are aware it is wrong, and the feedback is not as good as expected, well, that is also good, so then you will know what you don’t know; P5:It’s important to experience a crash and burn station, so that you learn to pick yourself up and keep going with the rest of the exam, so you don’t blow the whole thing. P6: So getting from 20 pages in Greenberg to a very nice, sophisticated, compact, concise delivery in 2 minutes, that’s difficult, you know, no one teaches you that. It’s trial and error	Learning from failures	Challenge	**Stress**
P3: very nice to see them push different people different amounts until you break; that really stretches you; put on the spot; competitive comparison P4: Step up to the plate;	Rising to challenge and competition		
P3: one has to practice thinking through the case in a limited period of time under stress	Time pressured		
P1: Threatened; embarrassment; P2: Safety in numbers; under fire; people will call in sick for vivas, because they don’t want to look stupid; relieved I didn’t have to go; grilled; the wrong kind of pressure; anxiety; (lack of) control; P3: exam set up where the time is tight, there is a lot of pressure, you are getting marked P4: Some think they will be embarrassed even if their line of thought is good. P6: There is so much hype about the exam, and they see it in the senior trainees. It’s a scary experience.	Pressure as a negative factor	Anxiety	
P4: ultimately it comes down to crunch time where you have to sit down and read about it, because tomorrow will be another embarrassing day. So there is a combination of coercion and continuous education. It works both ways. I know myself, I can be quite lazy and if I am not pushing myself then I expect others to push me. P5: I used to hate the mock viva when it was first introduced when I was ST5, even though I knew it was a good thing to do, to start motivating my exam preparation.	Pressure as a motivating factor		
P1: Split into junior and senior registrar groups is sensible; P1: Useful if close to exam P2: personally would not want to go for a mock viva until I was ST5 or 6, and know that I was really confident in what I knew; I think at an ST3 or 4 level you are constantly going to think I don’t even know what I’m talking about; (regarding the positive aspects of coaching) then you are not being tested on the content, they are testing you mainly on the delivery P2: If you don’t know the content, you’re stuffed; you wouldn’t go to an MRCS mock viva without knowing the content; that would be a waste of everyone’s time; if you don’t know the content, you’re going to fail. P4: In order to have an adequate process, you have to have content. So there is no point going through the process. I mean there is, but it is not as good as when you have appropriate context where it actually reflects the real time exam, and then you have the process on top of that. P5: It all feels a bit cold turkey when you are doing your first run through the mock viva as a very junior trainee, and I think trainees might benefit from a more collaborative approach, learning from each other, then just being tested on their individual knowledge at an early stage P6: if you are struggling through the basics, then they won’t ask you tougher questions, and if they won’t ask you tougher questions you won’t get marked higher; I think if there is a limit to what they know they are going to be asked about, that would help.	Limited knowledge limits educational value of mock viva process	Content vs Process	**Differentiation strategies for junior and senior trainees**
P1: Level doesn’t dictate performance P2: Communication skills; all to do with the process P3: your knowledge has improved a little bit each time and you can progress to the next level of questioning on the same case. P5: Knowledge is important, but you are being judged more on your thought processes, being able to prioritise clinical information and make reasoned sensible decisions. P6: some things you just need to actually have a go at, before you have any idea about what works and what doesn’t, and I think the FRCS is one of those things; *(talking about issue of junior trainees level of knowledge influencing participation)* That’s what I’ve been trying to turn on its head, because the questions start from the basics P6: And so if they know they are not going to be grilled over something very advanced, and can easily answer 70-80% of the questions asked I think that will alleviate the stress. It’s not just about the facts, it’s about the delivery.	Engaging with the viva process is possible and necessary at the early stages of training, independent of level of knowledge	Content vs Process	**Differentiation strategies for junior and senior trainees**
P1: Level doesn’t dictate performance; can learn a lot from senior people who know a lot but answer badly. P4: one has to practice thinking through the case in a limited period of time under stress	Paradox of poor delivery in context of good knowledge		
P2: that type of coaching is the kind of things trainees do before consultant interviews; coaching is constructive and will teach you the skills and will let you to practice the skills too	Support for coaching	Coaching for junior trainees	
P4: is it actually representative of reality, and who is going to be included in this privileged group or non-privileged group? I don’t know. And where do you draw the line? It creates a bit of an artificial situation I think; Why would you do that? It’s all supposed to be a level playing field.	Against coaching		
P1: Teaches you how to behave; Practicing acting skills	Learning the Hidden Curriculum	Learning opportunity	**Assessment versus Learning**
P1: Doesn’t help learning content per se; Feedback not specific enough on content P3: but in terms of content, I think that’s picked up at work or when you are reading or whatever, and this is your opportunity to apply that knowledge you have	Practice rather than learning		
P1: People don’t think there are learning something new - they see it as them just telling things to people; See it as a test of knowledge; analogy to assessment is off-putting	Assessment detracts from the learning opportunity	Assessment	
P2: it is what it is. It’s a group of consultants who ask you questions on set content.	Primary aim is assessment		
P2: actually pushes you to know the content P5: Ours is so informal, no one really prepares for it or treats it like a real exam. P4: the more questions you get the better it is *(for learning content)*	Assessment promotes learning		
P2: like a job interview as your viva P5: The only long term consequence of participating is how your performance is viewed by your contemporaries and consultant colleagues, which may have some implications for future training.	Implications for future training	Judgement	
P3: I see it as a chance to shine in front of people you work for or you are going to work for; an opportunity for you to show off in a way, that you probably don’t on a regular basis	Opportunity to demonstrate competence		

## Appendices

**Appendix 1.**

Neurosurgical Trainees’ Perspectives of the ‘Mock Viva’

A. **Schedule for individual interviews with pre-FRCS trainees**

Location: Neurosurgical Seminar Room

Time: Thursday 13:00

**Introduction**

• Thank participants for attendance and confirm informed consent
• Reiterate aims and objectives of study

**Questions and prompts**

1. What do you think are the advantages to you of the ‘mock viva’?
2. What do you think could deter some trainees from attending the ‘mock viva’?
3. In what ways do you think it may be helpful for future FRCS preparation?
4. Would you make any changes to the mock viva, and if so, why?

**Close** Debrief and thank participants.

B. **Schedule for individual interviews with post-FRCS trainees**

Location: Neurosurgical Seminar Room

Time: At the convenience of participant

**Introduction**

• Thank participant for attendance and confirm informed consent
• Reiterate aims and objectives of study

**Questions and prompts**

1. What do you think are the challenges of preparing for the oral component of the intercollegiate FRCS examination?
2. In what specific ways do you think the mock viva is helpful for FRCS preparation?
3. Do you think the educational value of the mock viva lies in the *process* or the *content* elements of exam preparation?
4. What do you think could deter trainees, particularly junior trainees, from attending the ‘mock viva’?
5. What strategies could help alleviate these deterrents and increase levels of engagement and the educational value of the mock viva?
6. Do you think there is a role for coaching junior trainees in viva preparation?
7. Do you think there is a role for separating the *process* and the *content* elements of the mock viva activity for junior trainees?

**Close** Debrief and thank participants.

**Appendix 2.**

**Neurosurgical Trainees’ Perspectives of the ‘Mock Viva’**

**Information Leaflet for Participants**

You are being invited to take part in a research project. Before you decided it is important for you to understand why the research us being done and what participation will involve. Please take time to read the following information carefully and discuss it with others if you wish. Ask me if there is anything that is not clear or if you would like more information. Take time to decide whether or not you wish to take part. Thank you for reading this.

**What is the project’s purpose?**

I am carrying out this study as part of an MSc Medical Education project to explore the educational value of the ‘mock viva’ to neurosurgical trainees.

**Why have I been chosen?**

Neurosurgical trainees within the North Thames Deanery, from ST1-8, will be invited to participate in this study.

**Do I have to take part?**

It is up to you to decide whether or not to take part. If you do decide to take part you will be given this information sheet to keep and will be asked to sign a consent form. You can withdraw at any time without giving a reason and without it affecting any benefits that you are entitled to.

**What will happen to me if I take part?**

You will be invited to attend a focus group with other trainees/individual interview, which is anticipated to last approximately 30 minutes. Questions for the focus group/interview will be structured around the following objectives:

• To establish whether neurosurgical trainees value the mock viva as an annual formative assessment.
• To find out if post-FRCS examination trainees consider the mock viva to be useful preparation for the summative assessment.

**What are the possible disadvantages and risks of taking part?**

Whilst there are no anticipated disadvantages to taking part, some trainees may consider discussing formative or summative examination performance to be personal or sensitive information and may prefer not to participate.

**What are the possible benefits of taking part?**

It is anticipated that this work will contribute to our understanding of the advantages and limitations of this type of formative assessment and may identify areas for improvement.

**Will my taking part in this project be kept confidential?**

Audio recordings from the focus group/interview will be transcribed and anonymised. All the information that we collect about you during the course of the research will be kept strictly confidential. You will not be able to be identified in any ensuing reports or publications.

**What will happen to the results of the research project?**

The results from this study will be used in a written assignment for my MSc Medical Education degree and will be shared with the Neurosurgical Training Programme Director for North Thames. The data collected during the course of the project might be used for additional or subsequent research.

**Contact for further information**

Grainne McKenna

gsmckenna@gmail.com

**Appendix 3.**

**Neurosurgical Trainees’ Perspectives of the ‘Mock Viva’**

Informed Consent Form for Participants

**Researcher:** Grainne McKenna

**Project for Degree:** MSc in Medical Education

**Please complete this form after you have read the Information Sheet about the research.**

Thank you for your interest in taking part in this research. Before you agree to take part, the person organising the research must explain the project to you.

If you have any questions arising from the Information Sheet or explanation already given to you, please ask the researcher before you to decide whether to join in. You will be given a copy of this Consent Form to keep and refer to at any time.

**Participant’s Statement**

I agree that:

• I have read the notes written above and the Information Sheet, and understand what the study involves.
• I understand that if I decide at any time that I no longer wish to take part in this project, I can notify the researchers involved and withdraw immediately.
• I consent to the processing of my personal information for the purposes of this research study.
• I understand that such information will be treated as strictly confidential and handled in accordance with the provisions of the Data Protection Act 1998.
• I agree that the research project named above has been explained to me to my satisfaction and I agree to take part in this study.

Signature: Date:      

**Appendix 4.**

**Thematic analysis of coded data:**

**Appendix 5.**

Transcripts of Interviews

Pre-FRCS exam participants

Participant 1 - ST3

Participant 2 - ST3

Participant 3 - ST4

Post-FRCS exam participants

Participant 4 - ST8

Participant 5 - ST8

Participant 6 - ST8

Participants’ responses are coded P1, P2, P3, P4, P5, P6 respectively.

The questions and responses from the interviewer are documented in italics after the code - *I:*

**Participant 1**

JP ST3

I: What do you think are the advantages to you of the ‘mock viva’?

P1: It helps you realise the structure of the assessment and it gives you insight into the marking scheme. You get practice in formally answering questions about topics you have only ever spoken about informally. One can have a lot of knowledge, but need to be able to structure your answers in a more time pressured and rigid atmosphere.

You can learn a lot from listening to other people giving answers. I’ve been surprised how bad some of them were, even though they knew a lot, they didn’t come across well because of lack of fluidity.

You can also imagine yourself as an examiner, which sounds awful. But it’s not awful.

Useful to realise how important it is how you come across to the examiner.

I: What do you think could deter some trainees from attending the ‘mock viva’?

P1: People don’t want to look stupid, especially if not formally prepared, and so they can be anxious. It can be disconcerting to realise how little you know, which is a good thing, but might put people off. It can be easier to go into denial and sink into your comfort zone, but the mock viva forces you into acknowledging things you don’t know.

I: In what ways do you think it may be helpful for future FRCS preparation?

P1: Ultimately I think it’s a good thing, but the stress of it all puts you off. It shatters your delusions!

I: Do you think trainees see it as a learning experience?

P1: People don’t think there are learning something new - they see it as them just telling things to people. I think the main benefit is practicing ‘acting skills’.

I: Anything else you think puts people off?

P1: Looking stupid in front of colleagues.

I: Do you think people are more worried how they look in front of their contemporaries or their bosses?

P1: Colleagues.

I think there is also a lack of understanding of the format of the mock viva and the analogy to assessment is off putting. It draws attention how little you know and being in denial -

It should be positive thing, but we are not machines - we live in element of self-denial. We want to feel capable, knowledgeable, and it is human instinct to want to avoid scenarios where this is threatened.

I: Would you make any changes to the mock viva, and if so, why?

P1: I don’t think it is long enough for you to get effective feedback, but it is well structured

It should be as close as possible to the real thing - must be realistic to be of benefit.

It should be exactly like doing the exam - that’s really useful.

I: Do you prefer to do it in pairs or on your own?

P1: I would prefer to do it on my own - so not embarrassed in front of peers.

I: More formal or less formal?

P1: More casual mock viva - is useful, but equally, to do it properly, it needs to be realistic. You could almost do it two ways, casual versus real thing - but both are better when you have a certain level of knowledge.

I: Any thoughts on how it might be changed for different levels of training?

P1: At an earlier stage - small group environment would be less threatening and give more opportunities to learn from others.

I: How frequently should it be done?

P1: At least once a year - argue twice a year would be even more useful.

I: Who with?

P1: Other registrars, mix of levels is fine. The level doesn’t dictate performance - can learn a lot from senior people who know a lot but answer badly. Split into junior and senior registrar groups is sensible.

I: Do you think there is educational value in learning content, or is it more about the exam process?

P1: It helps you learn how to behave and answer, but doesn’t help learning per se. Helps to identify that there is more you need to know, but the feedback on content is not specific enough. All in all, it is an artificial scenario - it doesn’t reflect real life - it’s only learning for the exam.

**Participant 2**

AA ST3

I: What do you think are the advantages to you of the ‘mock viva’?

P2: Did viva as an ST2. Getting to know the kind of level of content or level of knowledge that is required for the viva. Getting reacclimatised to being vivaed. Those are the two main things to be honest.

I: So how do you think it helps you find out about the content?

P2: Because you assume that the consultants who are asking you the questions have an idea of the level of content required for FRCS, because they may have been examiners, or they may run these courses regularly.

I: So they may ask you the question, but how do you find out about the content that is required?

P2: Yeah, the content and the way they ask the questions, because you don’t know if the way you are vivaed for FRCS is the same as the way you are vivaed for MRCS for example so it’s good to know whether it is the same or whether you have to start learning communications skills as well as the content that goes with it to an extent.

I: How do you think you learn those things from the viva process, if you’ve never done it before?

P2: It is more of an eye opening thing than anything else. I think it’s just knowing how you need to prepare for the upcoming FRCS exam. Doing it as an ST2 is not so bad, because if you’ve recently passed MRCS, then you know roughly how to answer, which makes it easier in a way, and then it is just the content that is going to make you feel not confident, which will impact on your performance.

I: When you did it as an ST2 did you do it in pairs?

P2: I did it with another ST2.

I: What that a good thing?

P2: Yes. Safety in numbers.

*I: Did you learn anything from them, or from observing them being* vivaed *do you think?*

P2: I learned that my level of knowledge was not as good. It can either boost your confidence or knock your confidence if you feel you are not on par with people in your same peer group or year.

I: Would you have preferred it to have been done separately, or perhaps with someone more senior?

P2: I don’t think it makes a difference at that level. It depends on their knowledge level and it depends on how confidence you are both feeling. If you are not confident in your own knowledge and communication skills, it doesn’t matter who you’re with, you are going to feel like you are under fire and you are not going to cope well.

I: Do you think you maybe have the opportunity to learn from someone who does it well or someone who performs badly?

P2: I think with regards to communication skills, yes, but probably not with regard to content.

I: Yes, you either know it or you don’t. That’s interesting, because that’ something that JP thought as well, that it was entirely not to do with content, and it was all to do with process.

P2: Absolutely.

I: And learning that skill set of how you come across.

P2: Absolutely. Because, I was asked about pineal tumours, and I know absolutely nothing about pineal tumours, but you have the MRCS viva system, you have a way of trying to bullshit your way through.

I: It is interesting though, how one could run the handover meeting like a viva process on a more regular basis there might be scope for learning content?

P2: If the handover meeting was run as it is in some other units in London, the viva would not be an issue, because you are so used to being under pressure, and because you are used to being examined in a group as well, you have the added pressure of not wanting to look stupid in front of your peers and your consultant body. So it actually pushes you to know the content anyway and then actually a lot of that training is done more on delivery of content than anything else.

I: So what you’re saying is, our annual mock viva is all quite stressful because we don’t do it in practice that often?

P2: Yes, I think people will call in sick for vivas, because they don’t want to look stupid, essentially.

I: What do you think could deter some trainees from attending the ‘mock viva’?

P2: So what specifically would deter people from coming? So I know for a fact. So I couldn’t go to the mock viva, because I had to cover an on-call when someone was off sick. I was relieved that I didn’t have to go.

I: That’s interesting, it is certainly my feeling that the turnout is shockingly low, for what is a fantastic resource in the context of how little opportunity there is generally speaking. I certainly never had mock viva experience until ST6, and I heard about how they do it every single year in other units, up North in particular, and how they are sort of groomed for the exam process. And equally, how they have it nearly every day in other places, and I think the lack of preparation for it on an almost continuous basis makes it very stressful.

P2: I think the King’s model worked well because the SHO group were vivaed in the morning meeting separately to the registrar group, so a lot of us were trust grade SHOs and we were geared towards National Selection vivas, and we would practice those types of questions over and over again, and actually, they had a very high pass rate for national selection. And it was the same the registrars going in for FRCS - they were grilled in a very different way, and they did very well.

I: In what ways do you think it may be helpful for future FRCS preparation?

P2: I think it’s good to gauge how much preparation you need to do. It’s mainly that.

I: In terms of content or process?

P2: I don’t know. I think it’s a mixture of both. I think for me it would be more delivery because I wouldn’t be confident in my delivery until my content was fine, so I personally would not want to go for a mock viva until I was ST5 or 6, and know that I was really confident in what I knew, because then I would be able to sit down and make sure I passed the exam.

I: The problem is, you never are until right at the end! But I felt the same way.

P2: I think at an ST3 or 4 level you are constantly going to think I don’t even know what I’m talking about, so how can I sit there and persuade two or three consultant neurosurgeons that I know what I’m talking about.

I: Do you worry about how you come across to your peers or your consultants?

P2: 100%. Both. Both.

I: Yes, this is a recurrent theme. Would you make any changes to the mock viva, and if so, why? So what do you think we could do to make it better. Because it seems there are lots of potential advantages. It is almost a necessary part of exam preparation. So wat could we do to make it a more positive experience and less intimidating? Or to increase the educational impact of the viva?

P2: The way that it’s set up at the moment, I don’t think there is much you can do, because it’s a mock viva session, it is what it is. It’s a group of consultants who ask you questions on set content. If you don’t know the content, you’re stuffed.

The feedback sessions and debrief sessions are good.

I: You mean at the end of each station?

P2: Yes, rather than waiting till the very end.

I: Yes, I think so too.

P2: I honestly don’t think there is much scope for improvement the way it stands. Unless the consultants could be brought in from outside. Consultants that you don’t know, who you are not meant to be working with or for. For example if you are going to CXH in August and you have Mr Tsang asking you questions on spinal trauma, and you don’t know anything about spinal trauma, you are inevitably going to be leading on the back foot, and not do your best.

I: Yes, I felt similarly. It’s a difficult one though, isn’t it, because it sounds like being under pressure is key to preparation?

P2: Yes, but it’s the wrong kind of pressure, because if I was going for the FRCS I would know that most of the examiners I wouldn’t have net before, and it’s just delivery and content, and that’s something you can control.

I: So you don’t have to worry about the personal overlay.

P2: You don’t need the anxiety of knowing that - oh I’m going to work for that person and I don’t want to look like a fool. That’s something that you can’t really deal with. It’s almost like a job interview as your viva, and that’s no good.

I: Do you think that rather than it all being a formal formative assessment, that there could be a training aspect to it? Because I did a teaching session which in the end happened after the viva, about how to prepare for the viva. It was about how to break down the questions and how to develop a system, and how to concentrate on the key/take home points that the examiner is looking for in your response. Do you think that there is a role for that type of teaching, rather than just running people through the exam scenarios, do you think there is value in some form of coaching for it?

P2: Yes, because that type of coaching is the kind of things trainees do before consultant interviews. So it is the same process. There are going to be a lot of things that you are going to need to talk about in a way that you need to talk about them, that you’ve never talked about before, and for people at my level going for FRCS, it is the same as people of your level going for a consultant job.

I don’t think it necessarily needs to be done on the day of the vivas, I think that absorbs resources. It is something that could be done online courses.

I: Do you think that instead of running people through the mock viva, we should just replace that with coaching for viva scenarios?

P2: Yes.

I: And then have the mock vivas closer to the time of sitting the exam?

P2: Yes. It is good. Because I think the coaching is far more important than the viva itself, because at ST1-5 level, it is like being thrown in at the deep end, there is only so much you can learn from it, or you are just going to feel completely knocked by it, whereas the coaching is constructive and will teach you the skills and will let you to practice the skills too, as long as it is coaching that allows you to practice in a kind of mock viva. So you have a kid of mock viva, but you are not being tested on the content, they are testing you mainly on the delivery. And then that puts the onus on you to just learn the content in your own time knowing that you know how to answer the questions.

I: I think you have summed up the problem actually. I think the trainees don’t have the content in order to be able to practice the delivery, and the focus is on the content, rather than on practicing the delivery.

P2: Yes, you wouldn’t go to an MRCS mock viva without knowing the content; that would be a waste of everyone’s time.

I: The problem with deanery teaching until recently has been the didactic format, which means that you don’t really come away with that skillset.

P2: There is no role for didactic teaching in my opinion. Again, it is like going for a mock OSCE as a medical student. You will get x number of points for introducing yourself and washing your hands, but if you don’t know the content, you’re going to fail, and that’s that.

**Participant 3**

AC ST4

I: What do you think are the advantages to you of the ‘mock viva’?

P3: That is gives me an idea of my knowledge level in comparison to other people that I see being vivaed. It gives me an idea of what my knowledge level is like in comparison to what it’s meant to be at the end. And it also, I guess, you get into the habit of thinking in the framework that is necessary for the exam.

I: So we tend to do it with someone who is at a similar stage of training, which helps you compare with at least one of your peers. What part of the process helps you know what you need to know?

P3: The feedback at the end from the examiners is probably one of the most important bits of it. It is also very nice to see them push different people different amounts until you break, and so that really stretches you too. I remember things where I had never done it before, but I had heard about it and I talked about it as though I had done it.. but so if you know about something in theory, then you can say in theory, look, I’ve never done it before, but this is how I would do it.

I: Good, and do you think enough time is given for the feedback component in your experience? Because sometimes it’s a bit rushed.

P3: Yes.

I: So when you last did it, you were ST3, and you did it with someone at the same stage?

P3: Yes.

I: So, do you think there is learning value in terms of content, or is it just exam process?

P3: There are certainly scans that I have not seen outside of that sort of setting and I guess it’s useful to be acquainted to those sorts of cases, but in terms of content, I think that’s picked up at work or when you are reading or whatever, and this is your opportunity to apply that knowledge you have.

I: What do you think could deter some trainees from attending the ‘mock viva’?

P3: I guess if you don’t like being put on the spot, if you don’t like that competitive comparison. People don’t like the idea of having to I guess perform in front of their peers. I can see how that would put people off.

I: In what ways do you think it may be helpful for future FRCS preparation?

P3: I guess if you go regularly over a period over seven or eight years you develop an idea of what the line of questioning is like. I guess there is only a limited number of things you can get asked about in theory, so if you cover most of them in this rehearsal setting, then it is unlikely you will go to the exam and be phased by something you have totally not thought about at all.

I: And do you think there is sufficient scope within the current annual mock viva to achieve that?

P3: I think there is an element of repetition, because they are good cases, and it’s the same people running it, and so they will repeat the cases that they know about, so I guess having more variety would be more helpful, but even in the 3 years that I’ve done it, you realise that your knowledge has improved a little bit each time and you can progress to the next level of questioning on the same case.

I: So some of the junior trainees found the whole process incredible stressful, to the point where they didn’t go, or were relieved that they couldn’t go, and they found that they didn’t have enough confidence in their content to make it a really worthwhile exercise. They found it all to be quite negative because of the perception that they were showing themselves up in front of their colleagues or their bosses, be that present or future, and they felt they didn’t make the most out of the preparation for the process, which is what the aim of the mock viva. And some of the more senior trainees, having more insight into having been through the exam process, felt that in order to increase the yield of the mock viva process, that it might be helpful to separate the content from the process - for example, tell the more junior trainees, you will have 6 stations on x, y, z. in order to increase the value of the delivery process. They could prepare the content to practice the process. Another suggestion was that it might be helpful to have coaching for the viva at an earlier stage with practice as part of the coaching process.

P3: I can see the benefit of that kind of formal preparation, but then the longer you spend on one situation, the less variety you’ll get. I guess I see this sort of this as the exact opposite of what you described because I see it as a chance to shine in front of people you work for or you are going to work for, and so actually if they know that ‘this guy’s junior’, but see that you are able to deal with complications, or at least in theory you know about them, then that’s an opportunity for you to show off in a way, that you probably don’t on a regular basis on the wards or wherever.

I: And so actually if it’s a bit too prescribed you don’t have that opportunity to demonstrate your knowledge about the basics?

I was just telling you that because I wanted to do a focus group and I wanted your opinion on those ideas.

Would you make any changes to the mock viva, and if so, why?

P3: Maybe have it in different places with different examiners, so there’s a bit more variety in examining styles. Also then people that you are less close to and therefore a bit more like a real exam in terms of you not knowing the examiners, and them not knowing you.

I: So a bit more objective?

P3: Yeah, more like the real thing.

**Post-Exam
**

**Participant 4**

DD ST8

I: What do you think are the challenges of preparing for the oral component of the intercollegiate FRCS examination and in what specific ways do you think the mock viva is helpful for FRCS preparation?

P4: I think it’s essential and I still think that the more mock viva experience one has, the better the chances of passing. It is simply practice giving an answer and thinking through the cases. It’s extremely important when it comes to exam set up where the time is tight, there is a lot of pressure, you are getting marked, and so it is very important to practice the actual process. Not necessarily individual questions, yes, that’s also beneficial, the more questions you get the better it is, but even without it, one has to practice thinking through the case in a limited period of time under stress, and that is extremely important.

I: So do you think it is more about the process than it is the content?

P4: It’s both. Content is essential as well. In order to have an adequate process, you have to have content. So there is no point going through the process. I mean there is, but it is not as good as when you have appropriate context where it actually reflects the real time exam, and then you have the process on top of that. So you have to have both.

I: What do you think could deter some trainees from attending the ‘mock viva’?

P4: Well, I think there are multiple reasons to it. One is that the feel they may not be able to make that step up and give adequate, coherent answers, thus they feel embarrassed. And, this is a purely subjectively line of thought, however, I understand that. We all understand that moving from junior to more senior positions requires getting involved in this type of quizzing all the time. As far as I can remember, that was always a part of my training. So it is essential, that one has to put back this embarrassment, in inverted commas, and step up to the plate. So that’s one answer.

Another is simply that their knowledge is inadequate, and some are embarrassed by that. Some are worried about being embarrassed, because they think they will be embarrassed even if their line of thought is good.

I: But they still need to practice the process?

P4: Practicing the process and practicing this is essential, and it becomes more important as time progresses. It is important to stress now that time is tight, and in 2 or 3 more years they will be in more senior positions and the time wasted now is time wasted forever. So they will have to catch up fairly rapidly when the time comes at some point. And then it will be really stressful. SO I think these fears are subjective and they are completely irrational, but then all are fears are mostly irrational.

I: Do you think the process could actually help trainees learn content, or do you think it should be focused on the process of the exam only?

P4: I think it is both. I think you have to learn something, and at the same time be able to process that information and be able to deliver it adequately. So it’s a combination of both.

I think the best mock exams that I have had here, for example, are when my knowledge was pushed to the very limit, and when I could not produce coherent and adequate answers. Those exam scenarios stuck to my mind the best, so in fact, although it may not feel like it at that moment in time, you learn most from that and you might actually benefit the most from those.

I: Is that from your own perception that you knew what you didn’t know, or was that from feedback you received at the time?

P4: I think sometimes there were things I did not know, or questions I did not know how to answer, and so that’s great because you have to think ‘how am I going to answer this question I actually don’t know?’ And so there is a way to do that as well, being logical and coherent and produce some kind of answer that may give you a few marks, and that is important. But then again if you push to the area where you give wrong answers, and you are aware it is wrong, and the feedback is not as good as expected, well, that is also good, so then you will know what you don’t know. So it works both ways, you know, once you get over a certain mark, and your start learning things that are becoming more presentable, and you are getting to the pass mark, then at that point you will most likely be giving answers which are relatively coherent, but you will become aware of your ignorance in certain areas, but if you know less, then this awareness of ignorance is less likely to be there. But both are useful.

I: So last question, and I will just give you some of the ideas other people came up with to see what you think of them. Would you make any changes to the mock viva to increase levels of engagement and increase the educational value, particularly for the junior trainees? You have already said for the more senior trainees it is pretty much essential, but the more junior ones sort of feel that they don’t actually have enough content to practice the process, so of them, not all of them. Some of them like to show off what they know. Certain suggestions include giving advance warning of the topics to be covered, so they can prepare for it, so they are more focused on the delivery and it might increase the yield of the educational experience, and reduce the stress of not knowing what will come up. What do you think of that suggestion?

P4: It’s not a bad suggestion, but you come to the point where you have to think, is it actually representative of reality, and who is going to be included in this privileged group or non-privileged group? I don’t know. And where do you draw the line? It creates a bit of an artificial situation I think.

My personal feeling, and I think that it probably works the best, but sadly not routinely in this department. Daily questioning is essential. If you start doing is regularly, even coming in early to do so, after six months or so, you really start going into greater depth, and when the mock exam comes, there is no need to warn people about anything, because they know already. Common things are common. So if you do that consistently throughout six months, then at the end of six months, you can say we are going to wrap this up in a more formalised way.

I: Yes, that’s the ideal.

P4: The ideal is that every single person in that six months is reading something. Then it really doesn’t matter you know, what topics come up in the exam, because most of them will have come up in the morning meetings, and also, should have been read/studied all the way through. If you read half an hour a day, that’s plenty. Sadly we didn’t continue with that since the exam.

I: I guess sometimes the assessment forces you to learn, which is the good thing about it?

P4: Absolutely. So if you are quizzed about vascular supply, or about basic anatomy every morning and every morning you don’t give a coherent answer, then ultimately it comes down to crunch time where you have to sit down and read about it. Because tomorrow will be another embarrassing day. So there is a combination of coercion and continuous education. It works both ways. I know myself, I can be quite lazy and if I am not pushing myself then I expect others to push me.

I: One last thing. Do you think there is a role for taking more junior trainees, who perhaps haven’t done a viva and giving them training in viva preparation, or do you think we should just give then a dry run like everyone else?

P4: What kind of training?

I: Where you talk about the content in advance and break down the questions to consider what the examiner wants to hear in the answers?

P4: You could do, but it is the same problem of where do you draw the line about who gets special treatment and who doesn’t? Who is junior, who is not so junior, who is middle ground? I don’t know. It’s difficult. Why would you do that? It’s all supposed to be a level playing field.

**Participant 5**

PG ST8

I: What do you think are the challenges of preparing for the oral component of the intercollegiate FRCS examination?

P5: The viva exam is a performance. You are being judged by consultant colleagues based on this performance, and so how have to convince them that you are one of them. This is what I tell trainees when I give my talk on the exam.

Knowledge is important, but you are being judged more on your thought processes, being able to prioritise clinical information and make reasoned sensible decisions.

Most of this we do on a day to day basis, but the exam is a highly pressurised situation, and so you have to practice the ‘exam technique’ in order to perform well on the day.

I: In what specific ways do you think the mock viva is helpful for FRCS preparation?

P5: For me, the most helpful aspects were getting familiar with the mark scheme, gaining insight into the classical exam scenarios and then, towards the end, getting in the zone, or exam mode, where you are imagining it is the real thing, so that you are familiar with the format and the feeling of being put on the spot on the day. It’s important to experience a crash and burn station, so that you learn to pick yourself up and keep going with the rest of the exam, so you don’t blow the whole thing.

I: What do you think could deter some trainees from attending the ‘mock viva’?

P5: I used to hate the mock viva when it was first introduced when I was ST5, even though I knew it was a good thing to do, to start motivating my exam preparation. In some ways it wasn’t that bad, and I was reassured that some of my contemporaries performed similarly, but it was a big eye opener into how much I didn’t even know I needed to know about! The turnout is always quite low, so I expect others felt the same way, especially in the ST3-4 group.

I: Would you make any changes to the mock viva, and if so, why?

P5: We should have more continuous preparation for the viva, with more ‘hot spotting’ in handover meetings and so forth. It all feels a bit cold turkey when you are doing your first run through the mock viva as a very junior trainee, and I think trainees might benefit from a more collaborative approach, learning from each other, then just being tested on their individual knowledge at an early stage. It all depends on the quality of the feedback, and often trainees measure their own performance on the basis of comparison with just one of their peers, which isn’t really a useful benchmark for what they need to know long term.

Also, the viva performance doesn’t really carry any weight - there are no real scores or measure of performance relative to the whole cohort. In other units, you need to have been assessed to have passed a formative viva locally before you will be supported to apply to sit the FRCS, and so trainees take it very seriously. Ours is so informal, no one really prepares for it or treats it like a real exam. The only long term consequence of participating is how your performance is viewed by your contemporaries and consultant colleagues, which may have some implications for future training. So maybe it could be considered to be compulsory, and become a more reliable and valid formative measure of performance.

**Participant 6**

AE ST8

I: What do you think are the challenges of preparing for the oral component of the intercollegiate FRCS examination?

P6: First of all, remaining calm, because the mind-set is different from the written exam, and you are marked on how fluid and calm you are, and that comes across to the examiners straight away, and you can’t control that. You can hide behind paper in the written exam, but there’s nowhere to hide in the viva. You need to have a poker face.

I: So it’s about the delivery?

P6: Absolutely, delivery. Very important.

The second thing is being able to figure out what the examiner wants and give it to them in a nice package, so to speak. So understanding what they want from the question.

Number 3, being able to shift between different aspects of concepts in the middle of the exam. For example, being able to move from the indications for an MVD, and the anatomy, and the operative approach and then switching over to side effects of Baclofen, or ethics, absolutely.

I: So that’s flexibility?

P6: Yes.

And I think the fourth thing is being able to have system of thinking. So you are asked a question, this is probably more relevant to the clinical, but in the viva it is important as well, where you can filter out everything else that is being asked of you, and just go for the jugular.

I: It is interesting, because everything that you have described there is something that I think trainees lack insight into.

P6: Absolutely.

I: So even when they are going for the mock viva, they don’t even realise what it’s all about.

P6: Absolutely, totally.

I: Unfortunately.

Next question: In what specific ways do you think the mock viva is helpful for FRCS preparation?

P6: First of all, it simulates the exam and then going back to things like Top Gun and Red Flag - after the Vietnam War, they found that pilots who survived the first 10 missions were much more likely to survive the rest of the war. And that’s why they aimed to simulate the first 10 missions, that’s why red flag exists, top gun exists, air force bases in Nevada, and there are 10-15 basic missions everyone has to go through. So it’s like top gun or desert flag for neurosurgery trainees.

The second thing is there’s a wealth of information about any topic in neurosurgery and the viva teaches you how to condense that into relative things, the important things that you can deliver in 2-3 minutes. You know, it will be 20-30 pages in a book, but there are key points that examiners want you to touch upon and from those key points they will decide on whether you have a sound understanding of the topic or not. So getting from 20 pages in Greenberg to a very nice, sophisticated, compact, concise delivery in 2 minutes, that’s difficult, you know, no one teaches you that. It’s trial and error.

I: So do you think that the mock viva actually teaches you that though? (I’m playing devil’s advocate).

P6: I do, because after the mock viva you will get some feedback. If you did well, that’s fine, and

I: You mean you get feedback from your own reflection on how it went, and the feedback you get from the examiners?

P6: Correct, yes.

I: So you think that going through that process teaches you how to prioritise and condense?

P6: Exactly, so in the words of Duke Sampson, who is the head of the Department of Surgery at UHT in Houston, and he’s a vascular Neurosurgeon, and basically one of the hotshots, and he said, ‘Don’t aim to put the clip on the aneurysm and occlude it perfectly first time, the first application of the clip will show you what you need to do, and it guides the way for the second aneurysm application.

I: So it’s the first step in gaining insight into what you need to do to pass the exam?

P6: Exactly, some things you just need to actually have a go at, before you have any idea about what works and what doesn’t, and I think the FRCS is one of those things.

I: What do you think could deter some trainees from attending the ‘mock viva’?

P6: Fear. I mean the exam is comprehensive, it’s robust, but there is a system to it. If you know the system, you can pass. It’s limited information, it’s not infinite- they are only asking you about neurosurgery, not the stock exchange or anything else. There is so much hype about the exam, and they see it in the senior trainees. It’s a scary experience. Having done it I can look back and say it wasn’t that bad, but I think what deters trainees, it’s the fear.

I: It’s becoming obvious from speaking to the juniors that they don’t feel they have sufficient content in order to be able to practice the delivery. What do you think of that as a concept?

P6: That’s what I’ve been trying to turn on its head, because the questions start from the basics, so every question starts with something simple - glucose, or what hormones the pituitary secretes, and this is medical school stuff. So if you don’t get through that, you are not going to be asked whether SRS is a good idea for someone who has had craniotomy and resection of a growth hormone secreting tumour three times before. You are not going to get to that point. It’s step by step. And I experienced this, If you know your basic sciences well, then preparing for the viva becomes much easier, because you don’t have to worry about numbers or figures, or basic science, you worry about advanced concepts, and examiners love that, but if you are struggling through the basics, then they won’t ask you tougher questions, and if they won’t ask you tougher questions you won’t get marked higher.

I: Would you make any changes to the mock viva, and if so, why? What do you think would reduce the stress of the process for trainees and maximise the educational value of the mock viva for them?

P6: I think if there is a limit to what they know they are going to be asked about, that would help.

I: Oh, that’s a good idea! So help them prepare for it in terms of content. That’s a really good idea.

P6: So a PP presentation maybe, or a series of bullet points. We are going to ask you about CSF, where is comes from, what it’s made of, hydrocephalus. Really basic stuff and say this is for ST2,3 for example, ST 4,5 we are going to ask you about operative management.ST,8, we are going to ask you about pathological conditions involving CSF disorders - IIH, complications of procedures and so forth. And so if they know they are not going to be grilled over something very advanced, and can easily answer 70-80% of the questions asked I think that will alleviate the stress. It’s not just about the facts, it’s about the delivery.

I: Yes. Completely. And even with a more senior trainee, you could say - we will cover these 6 topics, they could do a bit of preparation, and they would get more out of it?

P6: So take away that element. That’s what I did for the viva, I took Elwell’s book, and for me, I prepared the answers.

I: Yes, but probably for 40-50 topics.

P6: Having said that, as an ST3-4 you have a lot of time to prepare, and there is a template you can learn and apply to everything.
