# Supplementary material for: Neurosurgery Trainees’ Perspectives of the ‘Mock Viva’
Source: MedEdPublish (2016). 2018 Nov 7;7:248. [Version 1] doi: 10.15694/mep.2018.0000248.1 (PMC10898496; doi:10.15694/mep.2018.0000248.1)
Supplement: Peer review history

# Reviewer report: Reviewer response for version 1

**DOI:** 10.21956/mep.19704.r29471

**Published:** 2019-06-04

**Authors:** Ken Masters

**Affiliations:** 1 Sultan Qaboos University

This review has been migrated. The reviewer awarded 4 stars out of 5 A useful paper examining Neurosurgery Trainees’ Perspectives of the ‘Mock Viva.’ The authors explain the overall concern, which is that poor performance in final qualifying viva voca may be due less to lack of knowledge, and more to a lack of confidence and performance in the examination. The authors set up a mock viva voca, and evaluated their students’ perception of it, especially in light of a low uptake of the opportunity.Some comments:• The layout of Table 1 is a little strange, as usually, one would move from the broad to the specific, so reversing the order of the columns in Table 1 might have been better.• I really do appreciate the fact that the authors have included so much supplementary information. The medium of online publishing allows this, and the authors have used it well.• Some of the questions are a little too leading, though, and it would have been better if they had been broader in their approach. Nevertheless, a great deal of useful information was obtained.• The paper does tend to over-emphasis the “performance” aspect a little too much. The authors should guard against tipping the scales too far in the direction of preparation for an examination instead of ensuring that the candidates will be the best possible surgeons.Still, an insightful and useful piece of work.

Reviewer Expertise:

NA

No decision status is available

# Reviewer report: Reviewer response for version 1

**DOI:** 10.21956/mep.19704.r29470

**Published:** 2018-11-14

**Authors:** Richard Hays

**Affiliations:** 1 James Cook University

This review has been migrated. The reviewer awarded 3 stars out of 5 Thank you for the invitation to review this paper. While the methods are appropriate, the results come as no surprise. Mock exams are popular with candidates, particularly close to the time of the 'real' exams, and they can be good preparation. Success depends on many factors, including how similar are the mock stations in design, content and process to the real thing, and the quality of feedback provided. This is of course true for all assessments. However, there is a risk that the mock OSCE defines the de facto curriculum and is part of an 'underground' assessment bank managed by students, when it is really just a small sample of potential clinical assessments. Some candidates complain that they did well in a mock OSCE but then less well in the real thing. Therefore, it is also not surprising that those who achieved fellowship look back positively ( after all, they passed and the mock OSCE may well have helped), while those prpearing are more anxious. Practice may therefore be better for gaining confidence in OSCE processes and feedback on progress. I worry about the recommendation to have daily viva practice sessions as this may strengthen a 'teach to the test' approach at the expense of learning what is needed to be a neurosurgeon.

Reviewer Expertise:

NA

No decision status is available

# Reviewer report: Reviewer response for version 1

**DOI:** 10.21956/mep.19704.r29472

**Published:** 2018-11-07

**Authors:** Felix Silwimba

**Affiliations:** 1 University of Lusaka

This review has been migrated. The reviewer awarded 5 stars out of 5 The abstract has clearly described the methods, analysis and conclusion. The study is educative and can be replicated in other disciplines of medical education. I have learnt a lot from the use of qualitative methods in medical education. I sincerely recommend it.

Reviewer Expertise:

NA

No decision status is available
